# Phenotypic and genomic characterization of tigecycline heteroresistance in carbapenem-resistant *Klebsiella pneumoniae*

**DOI:** 10.1128/spectrum.03079-25

**Published:** 2026-01-08

**Authors:** Xingchen Tao, Yulian Xia, Liping Zhong

**Affiliations:** 1 Department of Laboratory, Jiaxing Maternal and Child Health Care Hospital, Jiaxing, Zhejiang, China; 2 Affiliated Women’s and Children’s Hospital of Jiaxing University, Jiaxing, Zhejiang, China; 3 The First Hospital of Jiaxing, Affiliated Hospital of Jiaxing University, Jiaxing, Zhejiang, China; Universita degli Studi dell'Insubria, Varese, Italy

**Keywords:** carbapenem-resistant *Klebsiella pneumoniae*, tigecycline, heteroresistance, whole-genome sequencing

## Abstract

The presence of tigecycline heteroresistance in carbapenem-resistant *Klebsiella pneumoniae* (CRKP) can compromise the efficacy of antimicrobial therapy. This study aimed to investigate the phenotypic and genomic characteristics of Tgc-HR in CRKP clinical isolates. A total of 52 non-repetitive CRKP isolates, all tigecycline-susceptible, were collected from January 2021 to December 2023. Tigecycline heteroresistance was confirmed by K-B disk diffusion, E-test screening, growth curves, population analysis profiling (colony pattern analysis), and time-kill curve in this study. Protein fingerprinting clustering, WGS analysis, and bioinformatics were used to investigate the phenotypic characteristics and resistance genes, virulence factors, and plasmid profiles of the isolates. The heteroresistant subclones could proliferate at tigecycline levels as high as 16 mg/L, and their resistant phenotype stayed stable even after repeated passages without antibiotic exposure. Analysis of protein fingerprint clustering suggested that the heteroresistant subclones were derived directly from the original strain, not from contamination by an external strain. All isolates were identified as ST-11 through multilocus sequence typing. The genomic analysis showed that the isolates contained numerous resistance genes, especially the tetracycline efflux pump gene *tet*(A), β-lactamase genes (*blaTEM* and *blaCTX-M*), the aminoglycoside-modifying enzyme gene (*aadA*), and the efflux pump regulators *ramR* and *acrR*. These resistance genes were found to co-occur with virulence factors, such as the type 3 fimbriae (mrk-associated loci), based on genomic co-occurrence analysis (which involves assessing the presence of genes in close proximity or co-localization on the genome), suggesting that resistance and virulence together could heighten pathogenicity and make treatment outcomes more challenging in clinical scenarios. The plasmid typing results indicated that the isolates contained a multireplicon plasmid capable of carrying several resistance genes and closely resembling known high-risk resistant plasmids, aiding in the dissemination of resistance. In this study, we systematically investigated the phenotypic and genomic features of tigecycline heteroresistance among clinical CRKP isolates in China and demonstrated its stability and plausibly genetic basis and hinted at treatment implications. The findings are predictions based off of genomic data estimations and have not been confirmed by transcriptomic nor functional research. More work is required to validate these mechanisms.

**IMPORTANCE:**

This study provides crucial insights into the phenomenon of tigecycline heteroresistance (Tgc-HR) in carbapenem-resistant *Klebsiella pneumoniae*. It highlights the stability of heteroresistant subpopulations and their ability to persist without selective antibiotic pressure, complicating treatment outcomes. The research shows that Tgc-HR is linked to multiple resistance and virulence factors, suggesting a co-evolution of resistance and pathogenicity. This finding underscores the importance of monitoring for heteroresistance in clinical settings, as it may lead to undetected therapeutic failures. Additionally, the study contributes to understanding the role of plasmids in the spread of resistance, emphasizing the need for comprehensive genomic surveillance to inform better antimicrobial strategies.

## INTRODUCTION

*Klebsiella pneumoniae* (KP) is a major hospital-acquired pathogen, and the emergence of carbapenem-resistant KP (CRKP) strains is of particular concern (1). Global spread of genes encoding for transferable carbapenemase led to an increase of CRKP in different parts of the world. The CHINET surveillance data have shown that the isolation rate of CRKP from China rose from 2.9% in 2005 to 26% in 2023 (2). Tigecycline is considered a last resort therapeutic option for CRKP infections because of its activity against many multidrug-resistant Gram-negative bacteria (1, 3, 4). However, new resistant strains of CRKP have been recently reported, particularly given the emergence of tigecycline heteroresistance (Tgc-HR). The presence of resistant subpopulations in a largely susceptible bacterial population presents a significant challenge to the efficacy of therapy and patient outcomes (5, 6).

Heteroresistance represents an intermediate stage between susceptibility and full resistance, characterized by the presence of resistant subpopulations within an otherwise susceptible bacterial population. This would impact any therapeutic regimens because the resistant subpopulations are often not identified by routine susceptibility testing, with subsequent therapeutic failures (7–9). The primary mechanism of tigecycline resistance involves overexpression of resistance-nodulation-cell division (RND) family efflux pumps, which actively transport tigecycline out of the bacterial cell, reducing its efficacy. This is particularly evident in the synergistic action of AcrAB and OqxAB efflux systems (10–12). In addition to chromosomal mechanisms, plasmid-mediated tigecycline resistance, which allows for the horizontal transfer of resistance traits between bacteria, has also garnered attention. Studies have reported CRKP strains harboring mutated *tet* efflux pump genes with altered substrate-binding properties, enhancing tigecycline tolerance (13). Furthermore, high-risk CRKP clones, such as ST-11, which are frequently associated with increased virulence and resistance to multiple antibiotics, often co-harbor multiple replicon plasmids that carry both resistance genes and virulence factors. This co-habitation raises the risk of co-evolution between resistance and pathogenicity (14).

The epidemiological situation, molecular mechanism, and association between Tgc-HR and plasmids and virulence factors in CRKP are still unknown, and further investigations are warranted. The phenotypic detection and whole-genome sequencing were used in the present research, which revealed the potential of Tgc-HR and described the characteristics of resistant genes, plasmid types, and distribution of virulence genes of clinical CRKP isolates, which are very important for the reference of clinical resistance monitoring, molecular epidemiology, and optimization of antimicrobial treatment.

## MATERIALS AND METHODS

### Bacterial strains

This was a retrospective study that included 52 non-duplicate CRKP isolates susceptible to tigecycline isolated from clinical infection specimens of inpatients at Jiaxing Veterans Hospital from January 2021 to December 2023. Two KP isolates, RJ30 and RJ32, both tigecycline-susceptible, were used as parent strains. The tigecycline-resistant subclones, RJ30_R and RJ32_R, were derived from these parent strains. Bacteria identification was performed using matrix-assisted laser desorption/ionization time-of-flight mass spectrometry (MALDI-TOF MS; Bruker Daltonics, Germany), the minimum inhibitory concentration (MIC) was determined by the Vitek 2 Compact system (bioMérieux, France). Interpretation of the tigecycline susceptibility was performed according to European Committee on Antimicrobial Susceptibility Testing (EUCAST) guidelines (version 15.0, 2025) as follows: MIC ≤ 1.0 mg/L (susceptible), 2.0 mg/L (intermediate), and > 2.0 mg/L (resistant) (15). *Escherichia coli* ATCC 25922 was employed as a quality control strain to monitor the accuracy and reproducibility of the susceptibility testing.

### Reagents and media

Tigecycline susceptibility disks (15 μg), used for antimicrobial susceptibility testing, were purchased from OXOID. Mueller-Hinton agar (MHA) was obtained from Qingdao Hope Bio-Technology (Qingdao, China), and tigecycline powder (pharmaceutical grade) was purchased from MCE Company.

### Kirby-Bauer disk diffusion and E-test methods

Tigecycline susceptibility disks (15 μg) and E-test strips were applied to MHA plates inoculated with bacterial suspensions adjusted to the 0.5 McFarland standard, which ensures a standardized bacterial concentration for consistent susceptibility testing. After incubation at 35°C for 24 hours in an aerobic environment, inhibition zones were examined for the presence of scattered colony growth. Strains showing inhibition zone diameters within the susceptible range, but with isolated colonies appearing within the zone, were considered potentially heteroresistant, suggesting the presence of a resistant subpopulation.

### Population analysis profiling

As the gold-standard method for confirming heteroresistance, 0.5 McFarland bacterial suspensions (equivalent to a standardized bacterial concentration of approximately 1 × 10⁸ CFU/mL) were serially diluted 10-fold, and 100 μL of each dilution was inoculated onto MHA plates containing tigecycline concentrations ranging from 0.125 to 64 mg/L (twofold increments), chosen to assess the gradient of tigecycline resistance and heteroresistance in a clinically relevant range. Plates were incubated at 35°C for 48 hours in an aerobic environment, and colony counts were recorded. To assess the stability of heteroresistant phenotypes, single colonies were selected from the edges of inhibition zones, subcultured on antibiotic-free MHA medium for ≥2 weeks to ensure the absence of selective pressure, and subsequently retested for MIC and disk diffusion results. Each strain was tested in triplicate. We used *E. coli* ATCC 25922 and species-matched KP ATCC 700603 as quality-control strains to verify assay performance.

### Time-kill curve assays

Single colonies from Columbia blood agar plates were inoculated into fresh Luria-Bertani (LB) broth and adjusted to the 0.5 McFarland standard (approximately 1 × 10⁸ CFU/mL), ensuring a consistent and standardized bacterial concentration for the time-kill curve assay. Tigecycline was added at 4× the MIC of the parent strain to assess the bactericidal effect under high antibiotic concentration, simulating a therapeutic dose with sufficient antibiotic pressure. Bacterial suspensions were incubated at 35°C with shaking at 150 rpm to ensure proper aeration and facilitate bacterial growth during the assay. At 2-hour intervals for 48 hours, 100 μL aliquots of the bacterial suspension were plated onto MHA plates, spread uniformly, dried for 5–10 minutes to remove excess liquid, and then incubated at 35°C in 5% CO₂ for 48 hours. Each treatment was performed in triplicate to ensure reproducibility and reliable results. We used *E. coli* ATCC 25922 and species-matched KP ATCC 700603 as quality-control strains to verify assay performance.

### Growth curve assay (OD₆₂₀)

Overnight cultures were grown in LB at 35°C with 5% CO₂ to stationary phase. Cultures were diluted 1:1,000 in fresh LB and dispensed into 96-well plates (200 μL/well). OD₆₂₀ was recorded hourly for 12 hours using a microplate reader model; medium blanks were subtracted. Each strain was assayed in triplicate (*n* = 3 technical replicates per strain). Growth curves were generated in GraphPad Prism. From ln (OD₆₂₀) vs time, μ_max_ (h⁻¹) was obtained as the maximal slope, and AUC_0–12h_ (OD₆₂₀·h) was calculated by the trapezoidal rule. KP ATCC 700603 served as the quality-control strain.

### Protein fingerprint clustering

Protein fingerprint analysis, a technique used to identify bacterial strains based on the unique pattern of proteins they produce, was performed using Bruker MALDI-TOF MS. All spectra were processed and normalized using the manufacturer’s software (Biotyper, Bruker Daltonics), which involved baseline correction, peak detection, and intensity normalization to ensure accurate comparison between samples. Cluster analysis based on spectral similarity was conducted to group strains with similar protein profiles, and an MALDI-TOF Spectra (MSP) dendrogram was generated to assess the homology and evolutionary relationships between the strains.

### Whole-Genome sequencing and assembly

Raw sequencing data were error-corrected using Pbdagcon (https://github.com/pb-cdunn/pbdagcon) to correct for sequencing errors, which improves the accuracy and reliability of downstream assembly. High-quality circular consensus sequence subreads, which are generated by improving the accuracy of raw reads through multiple sequencing passes, were assembled using the Celera Assembler to generate draft genomic unitigs. Single-nucleotide corrections were then performed using GATK (https://gatk.broadinstitute.org/hc/en-us) and the SOAP toolkit to improve base-calling accuracy and correct any remaining sequencing errors.

### Genomic typing

Seven-locus multilocus sequence typing (MLST) was performed using *gapA*, *tonB*, *mdh*, *infB*, *rpoB*, *phoE*, and *pgi* to provide lineage context. For higher-resolution contextual assignment, we additionally queried the Institut Pasteur KP BIGSdb 629-locus cgMLST scheme; clonal group (CG), sublineage (SL), and KP species complex assignments were retrieved from the database. We used cgMLST solely for contextual classification rather than transmission inference given the phenotypic scope and small sample size.

### Genome annotation and resistance gene prediction

Genome annotation was performed using a combination of RAST (https://bv-brc.org/app/Annotation), Prokka (https://github.com/tseemann/prokka), and BLASTN (https://www.ncbi.nlm.nih.gov/), which were used together to provide comprehensive functional annotation, predict protein-coding genes, and identify homologous sequences. Open reading frame prediction, which identifies potential protein-coding regions in the genome, was carried out using ORFfinder (https://www.ncbi.nlm.nih.gov/orffinder/). Resistance gene prediction was conducted using ResFinder 4.5.0 (https://bitbucket.org/genomicepidemiology/resfinder/src/master/), which identifies known resistance genes, with confirmation against the Comprehensive Antibiotic Resistance Database (CARD) (https://card.mcmaster.ca/), which provides a reference for gene characterization and resistance profiles. Resistance gene abundance was calculated as normalized read depth, a method that normalizes the data for sequencing coverage, allowing for accurate comparison of gene abundance across different samples.

### Virulence gene analysis

Virulence gene prediction was carried out by TBLASTN alignment of genome sequences against the virulence factor database (VFDB) (https://www.mgc.ac.cn/VFs/), a comprehensive database of virulence factors in bacteria, to identify potential virulence genes based on sequence similarity. The data were analyzed using the online tool of the Majorbio Cloud Platform (https://www.majorbio.com/tools), which provides comprehensive bioinformatics tools for functional annotation and statistical analysis of gene sequences.

### Plasmid typing analysis

Plasmid sequences were identified by BLASTN alignment against PlasmidFinder (Enterobacteriaceae plasmid sequence database) to determine replicon types and analyze multiple replicon structures, as well as their homology with known high-risk resistance plasmids.

### Data processing

Raw OD₆₂₀ readings (0–12 hours) were exported and blank-corrected using the medium control on each plate. Values were time-ordered and screened for missing/duplicated time stamps; no smoothing was applied. AUC_0–12h_ was computed from blank-corrected curves by the trapezoidal rule and reported in OD₆₂₀·h. The maximum specific growth rate (μ_max_) was estimated from the exponential phase as the maximum slope of ln(OD₆₂₀, corrected) vs time across sliding windows of ≥4 consecutive timepoints; windows with OD₆₂₀ ≤0.02 (noise) or ≥0.8 (near-saturation) were excluded. For each isolate (RJ30, RJ30_R, RJ32, and RJ32_R) and the reference strain (KP ATCC 700603), metrics were calculated per biological replicate (*n* = 3) and summarized as mean ± SD.

## RESULTS

### Screening and confirmation of Tgc-HR

Among 52 isolates, four showed microcolonies or E-test trailing in the screen, and population analysis profiling (PAP) confirmed heteroresistance in two isolates (3.85%). Table 1 summarizes the screening pipeline and outcomes.

**TABLE 1:** Summary of tigecycline-heteroresistance screening among 52 non-duplicate, tigecycline-susceptible CRKP isolates^*a*^

Metric	*n*	% of screened
Total CRKP isolates screened	52	100
Isolates with microcolonies within inhibition zones (KB and/or E-test)	4	7.69%
Isolates confirmed heteroresistant by PAP	2	3.85%
Isolates selected for downstream experiments	2 (RJ30_R, RJ32_R)	3.85%

^*a*^ Denominator = 52 non-duplicate, tigecycline-susceptible CRKP isolates.

A comprehensive table summarizing the MIC values for key antibiotics in the strains included in this study is presented below (Table 2). These values reflect the resistance profiles of the parent strains (RJ30 and RJ32) and their corresponding tigecycline-resistant subclones (RJ30_R and RJ32_R). The table includes MIC values for carbapenems, tigecycline, tetracycline, new β-lactam/β-lactamase inhibitor combination antibiotics, and colistin, providing a detailed overview of the multidrug resistance (MDR) characteristics of these isolates.

**TABLE 2:** MIC of antibiotics for different isolates^*a*^^,*b*^

Isolate number	MIC (µg/mL)														
	IMP	MEM	ETP	TGC	TC	CAP	LEV	AMK	GEN	CFS	TZP	FEP	CRO	CAZ	COL
RJ30	≥16(R)	>8(R)	≥8(R)	1(S)	>8(R)	≥64(R)	≥8(R)	≤2(S)	≤2 (S)	≥64(R)	≥128(R)	≥32(R)	≥64(R)	≥64(R)	1(I)
RJ30_R	≥16(R)	>8(R)	≥8(R)	≥8(R)	>8(R)	≥64(R)	≥8(R)	≥64(R)	≥64(R)	≥64(R)	≥128(R)	≥32(R)	≥64(R)	≥64(R)	1(I)
RJ32	≥16(R)	>8(R)	≥8(R)	1(S)	>8(R)	≥64(R)	≥8(R)	≥64(R)	≥64(R)	≥64(R)	≥32(R)	≥32(R)	≥64(R)	≥64(R)	1(I)
RJ32_R	≥16(R)	>8(R)	≥8(R)	≥8(R)	>8(R)	≥64(R)	≥8(R)	≥64(R)	≥64(R)	≥64(R)	≥128(R)	≥32(R)	≥64(R)	≥64(R)	1(I)

^*a*^ IMP, imipenem; MEM, meropenem; ETP, ertapenem; TGC, tigecycline; TC, tetracycline; CAP, cefoperazone; LEV, levofloxacin; AMK, amikacin; GEN, Gentamicin; CFS. cefoperazone/sulbactam; TZP, piperacillin/tazobactam; FEP, cefepime; CRO, ceftriaxone; CAZ, ceftazidime; COL, colistin.

^*b*^ S, sensitive; R, resistant; I, intermediate.

### Detection of tigecycline heteroresistant phenotypes

**Fig 1:**
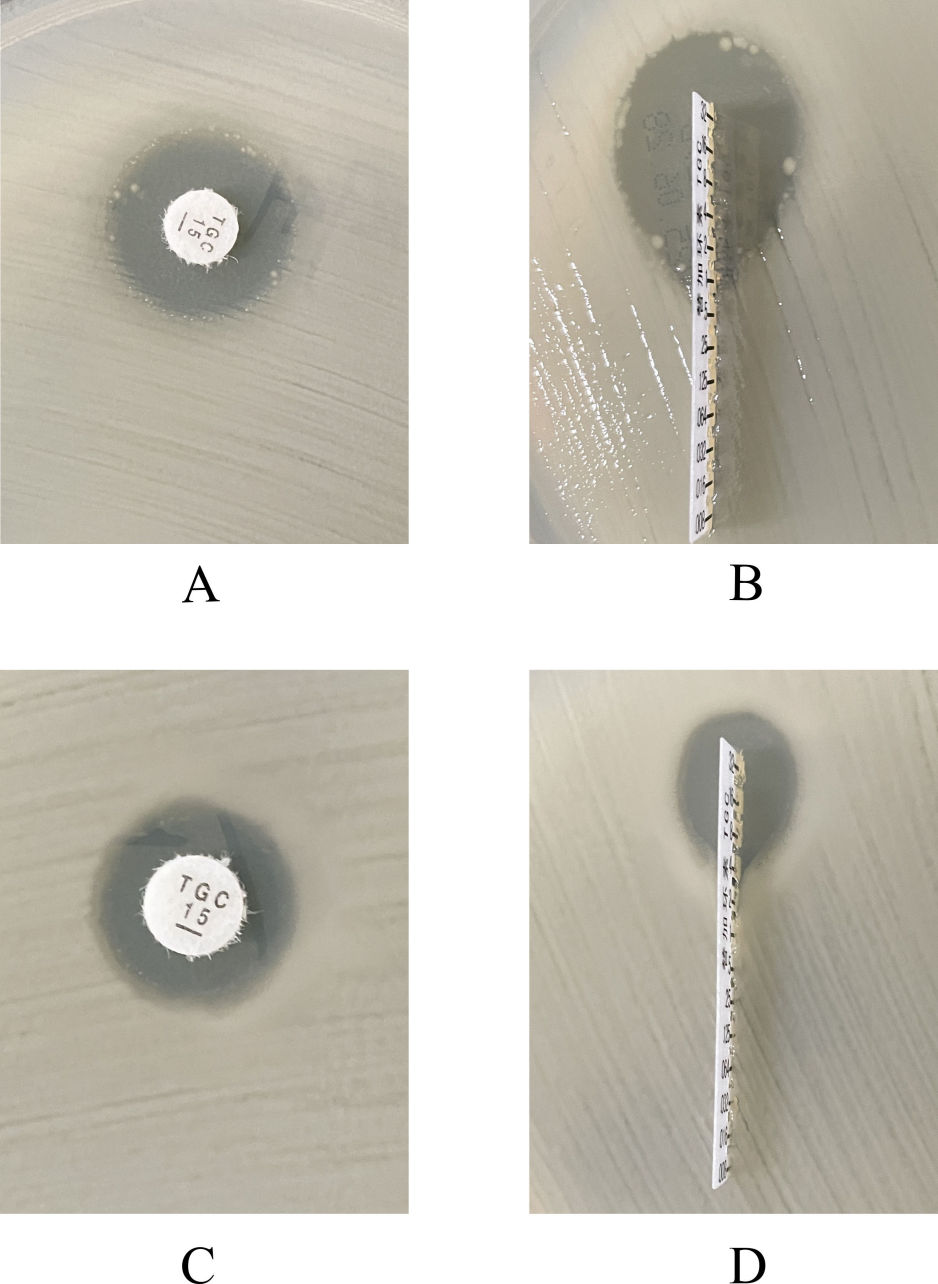
Detection of tigecycline heteroresistant phenotype by Kirby-Bauer disk diffusion and E-test. Representative isolate: parent strain RJ30 and heteroresistant subclone RJ30_R. (**A**) parent strain RJ30, Kirby-Bauer; (**B**) parent strain RJ30, E-test; (**C**) subclone RJ30_R, Kirby-Bauer; (**D**) subclone RJ30_R, E-test.

In Kirby-Bauer disk diffusion and E-test assays, some isolates exhibited scattered colonies within inhibition zones (Fig. 1A and B), a typical characteristic of heteroresistance, which indicates the presence of resistant subpopulations within an overall susceptible bacterial population. To verify stability, we analyzed two isolates with colonies present in the tigecycline inhibition zones; one single colony per isolate was picked (total two colonies) and re-tested after serial passaging on antibiotic-free medium for ≥2 weeks. The results showed significantly reduced inhibition zone diameters compared to the parent strains, with all measurements falling within the tigecycline-resistant range and no scattered colonies within the zones (Fig. 1C and D). This reduction in zone size further supports the stable expression of heteroresistance, as it reflects a shift toward a more resistant phenotype under antibiotic pressure. This phenomenon was consistent with the selective amplification of heteroresistant subpopulations under antibiotic selection pressure, where the resistant subpopulations survive and proliferate while the susceptible bacteria are inhibited by the antibiotic.

### Phenotypic confirmation of tigecycline heteroresistant strains

**Fig 2:**
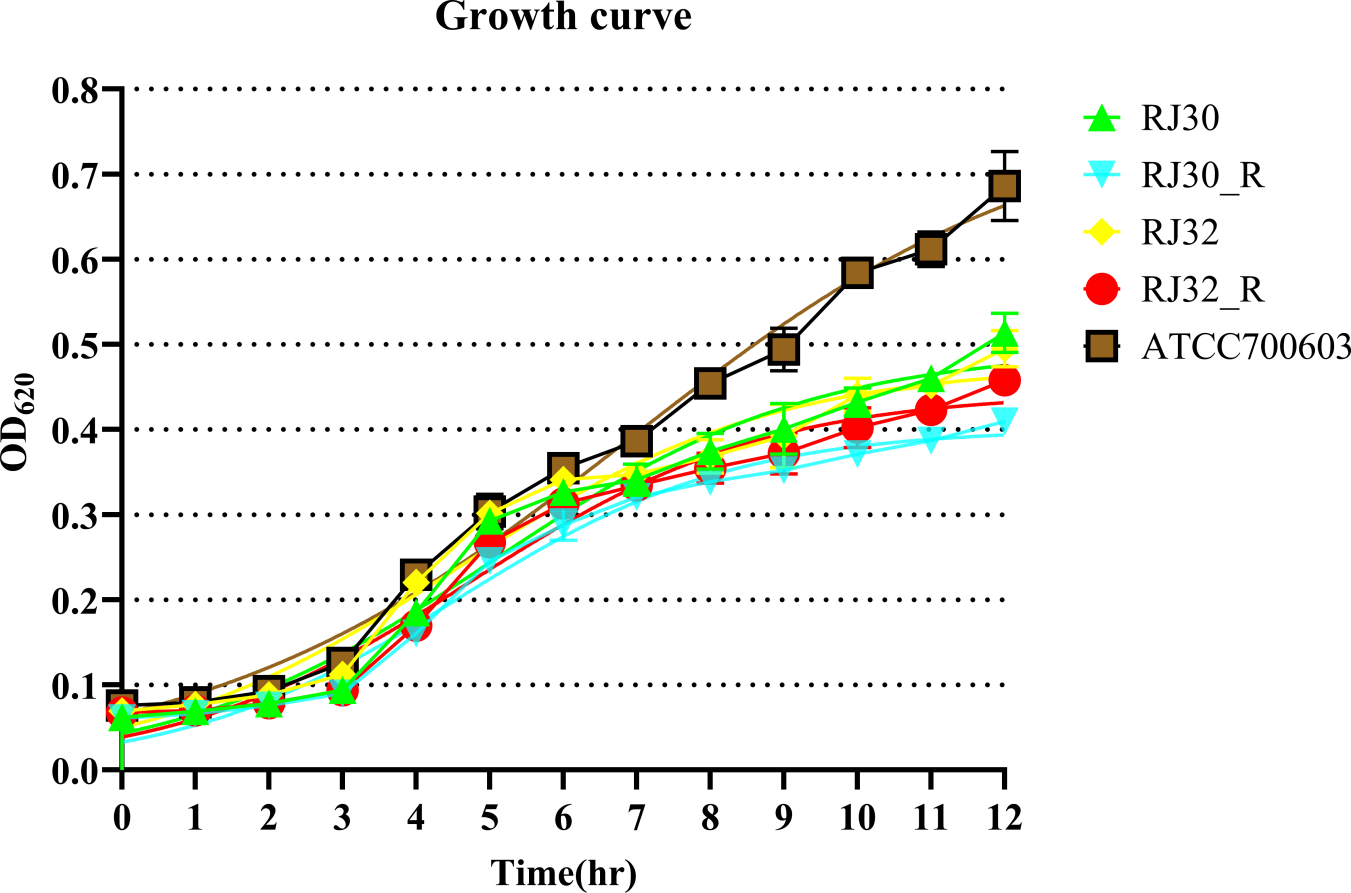
Growth curves of KP strains measured at OD_620_ (0–12 hours). Parent strains RJ30 and RJ32 and their heteroresistant subclones RJ30_R and RJ32_R were grown in LB; ATCC 700603 was included as a quality-control strain. OD_620_ was recorded hourly for 12 hours. Data are mean ± SD; *n* = 3 technical replicates per strain. Subclones exhibited growth kinetics comparable to their respective parents (2/2 pairs), consistent with μ_max_ and AUC_0–12h_ analyses (Materials and Methods).

Figure 2 shows OD₆₂₀ growth curves (0–12 hours) for two parental CRKP isolates and their heteroresistant subclones: RJ30 vs RJ30_R and RJ32 vs RJ32_R. KP ATCC 700603 was included as the reference (quality-control) strain. In 0–12 hours OD₆₂₀ growth-curve assays (*n* = 3 technical replicates per strain), the heteroresistant subclones showed growth kinetics comparable to their parental strains (2/2 pairs; 100%). Quantitatively, RJ30 vs RJ30_R: μ_max_ 0.401 **±** 0.023 vs 0.368 **±** 0.028 h⁻¹ (−8.29%); AUC_0–12h_ 3.343 ± 0.123 vs 2.931 ± 0.044 OD₆₂₀·h (−12.31%). RJ32 vs RJ32_R: μ_max_ 0.375 ± 0.024 vs 0.382 ± 0.016 h**⁻**¹ (+1.88%); AUC_0–12h_ 3.423 **±** 0.140 vs 3.141 **±** 0.118 OD₆₂₀·h (−8.23%). The QC strain ATCC 700603 exhibited μ_max_ 0.369 **±** 0.027 h⁻¹ and AUC_0–12h_ 4.097 ± 0.128 OD₆₂₀·h.

### PAP assays

**Fig 3:**
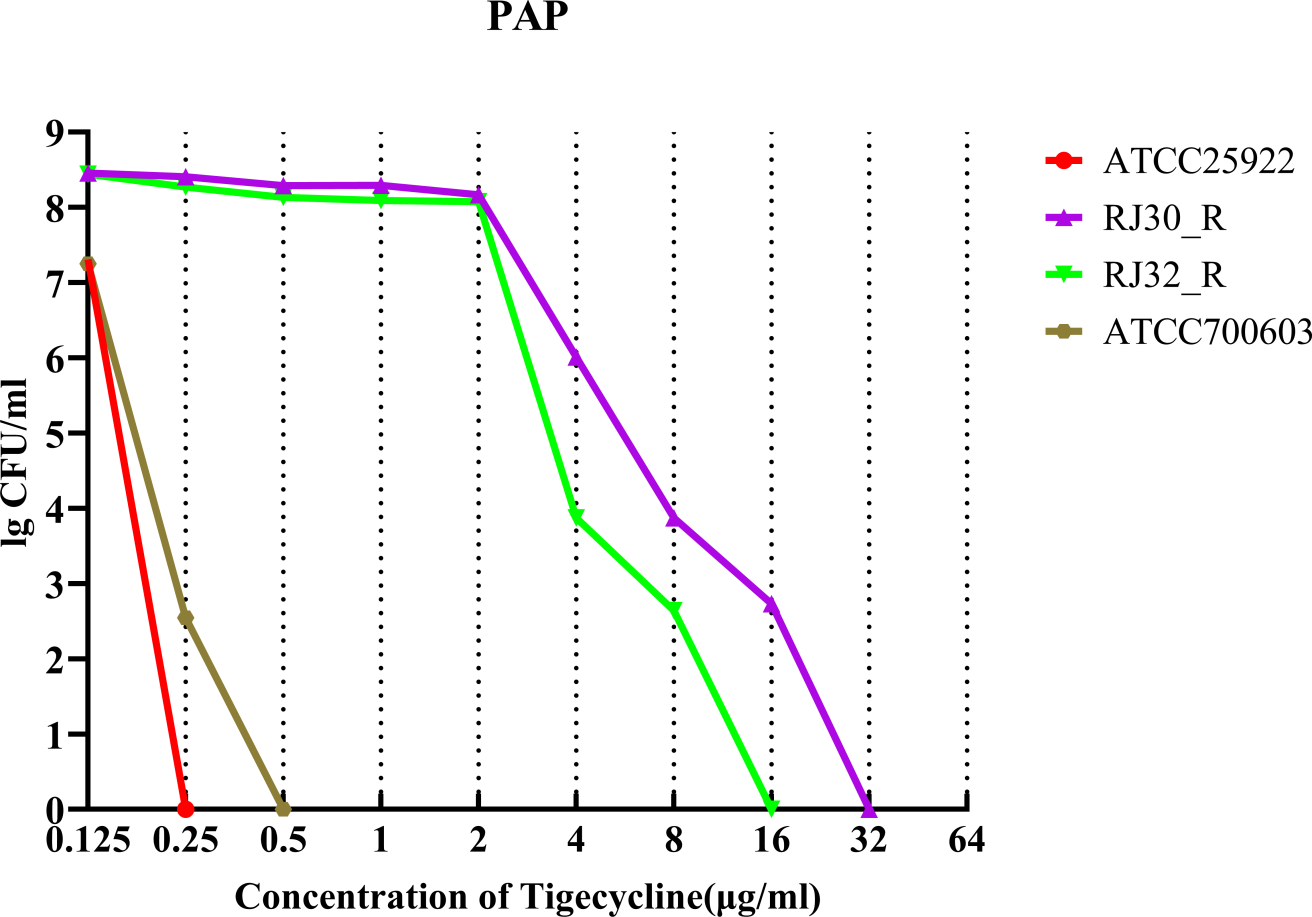
PAP test of tigecycline heteroresistant isolates. Both RJ30_R and RJ32_R demonstrated growth at tigecycline concentrations up to 16 mg/L. The results show bacterial counts at different tigecycline concentrations after 48 hours of incubation.

**Fig 4:**
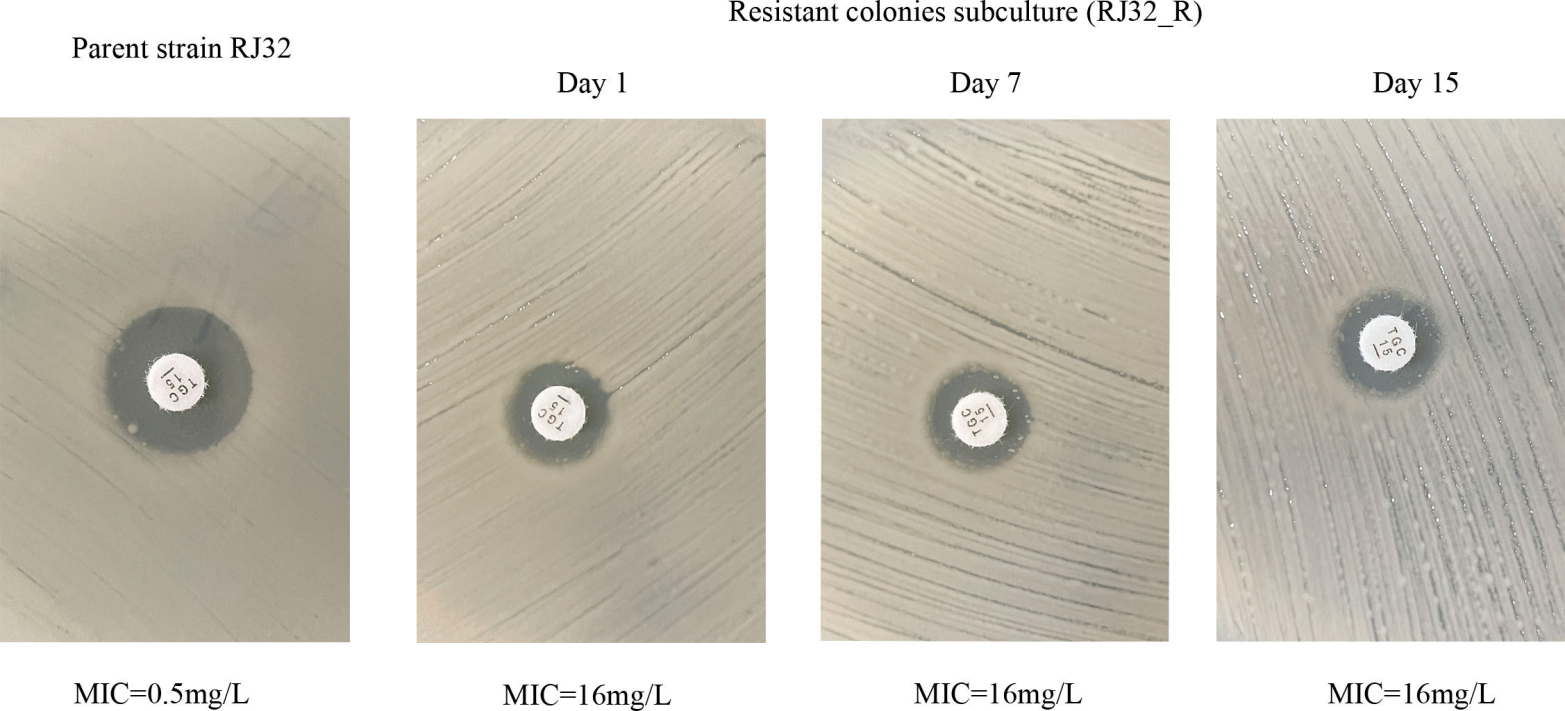
Stability of Tgc-HR in CRKP strains.

PAP assays confirmed that subclonal growth (growth of resistant subpopulations within an otherwise susceptible bacterial population) within tigecycline inhibition zones represented heteroresistant phenotypes (Fig. 3). Both RJ30_R and RJ32_R heteroresistant strains could grow at high tigecycline concentrations, up to 16 mg/L, which exceeds the typical MIC for susceptible KP strains. Additionally, after 15 days of subculture in antibiotic-free medium (which helps confirm the stability of the heteroresistant phenotype in the absence of selective pressure), all strains maintained stable tigecycline heteroresistant phenotypes (Fig. 4). These results further confirm the presence of Tgc-HR in clinical CRKP isolates.

### Time-kill curve assays

**Fig 5:**
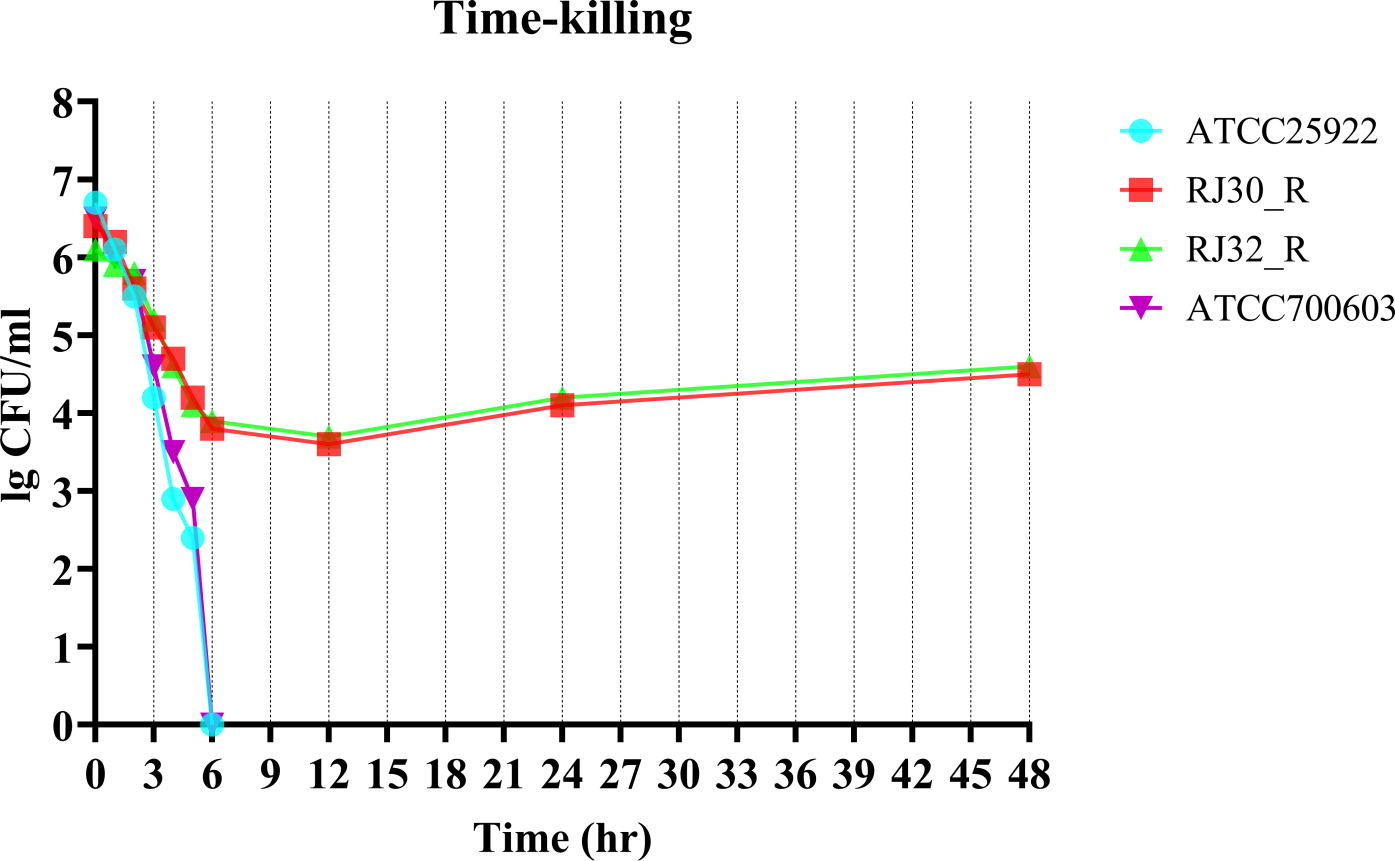
Time-kill curve of tigecycline heteroresistant strains. Bacterial survival was assessed over 48 hours in the presence of 4× MIC tigecycline. *E. coli* ATCC 25922 and KP ATCC 700603 served as the control strain.

In time-kill curve assays using 4× the tigecycline MIC (to induce strong selective pressure on the bacterial population), we observed growth patterns of two heteroresistant strains. Colony counts showed a moderate reduction (approximately 30%–40%) during the first 6 hours, followed by a slower decline (less than 20%) between 6 and 12 hours. However, after 24–48 hours of incubation, the strains demonstrated regrowth, suggesting that resistant subpopulations were able to survive antibiotic inhibition and resume growth. The control strain, *E. coli* ATCC 25922, which is known to be fully susceptible to tigecycline, was completely killed within 6 hours (Fig. 5). These data are consistent with survival and subsequent expansion of a less-susceptible subpopulation under tigecycline exposure; however, we cannot exclude the emergence or selection of additional adaptive variants during exposure (see Fig. 4 and 5).

### Protein fingerprint clustering analysis

**Fig 6:**
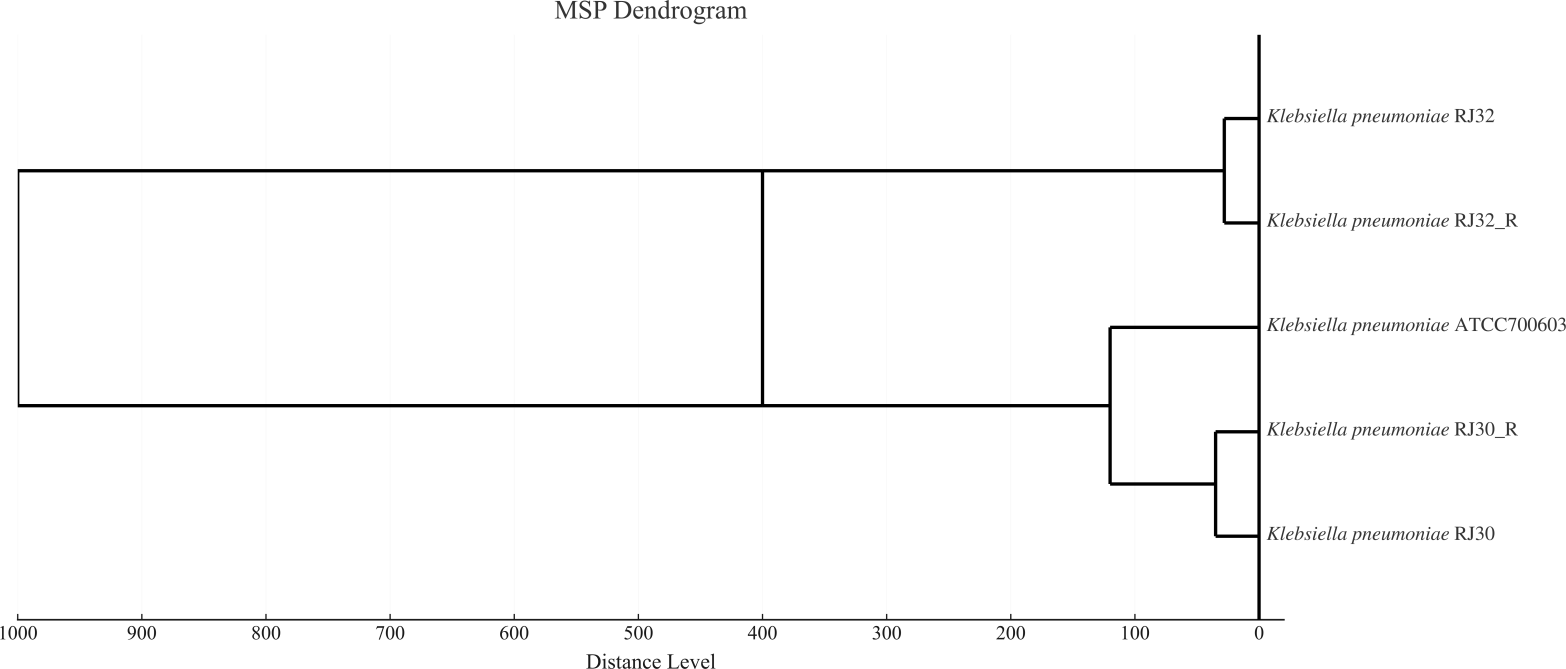
MSP dendrogram of KP strains showing hierarchical clustering based on mass spectrometry profiles. The distance level on the *x*-axis represents the degree of dissimilarity between strains, with closely related strains grouped together.

The results indicated that RJ30 and RJ30_R clustered into one group, suggesting they are highly similar at the protein level, with a low degree of divergence, while RJ32 and RJ32_R formed closely related branches, indicating high similarity but some degree of divergence in their protein profiles, suggesting high homology at the protein level between the two pairs of strains, which indicates genetic and functional similarity and helps to confirm their classification as closely related strains (Fig. 6).

### MLST typing

MLST analysis covered seven housekeeping genes (*gapA*, *tonB*, *mdh*, *infB*, *rpoB*, *phoE*, and *pgi*), which are commonly used for strain typing due to their conserved nature and ability to provide reliable genetic markers for bacterial identification. RJ30_R and RJ32_R exhibited 100% sequence identity at all loci, indicating that both strains are genetically identical at the loci examined. Both strains belonged to ST-11.

### cgMLST

Using the 629-locus cgMLST in the Institut Pasteur BIGSdb, both RJ30_R and RJ32_R were assigned to Kp1/CG11/SL258, consistent with their ST-11 MLST background and supporting placement within the same high-risk lineage.

### Resistance gene analysis

**Fig 7:**
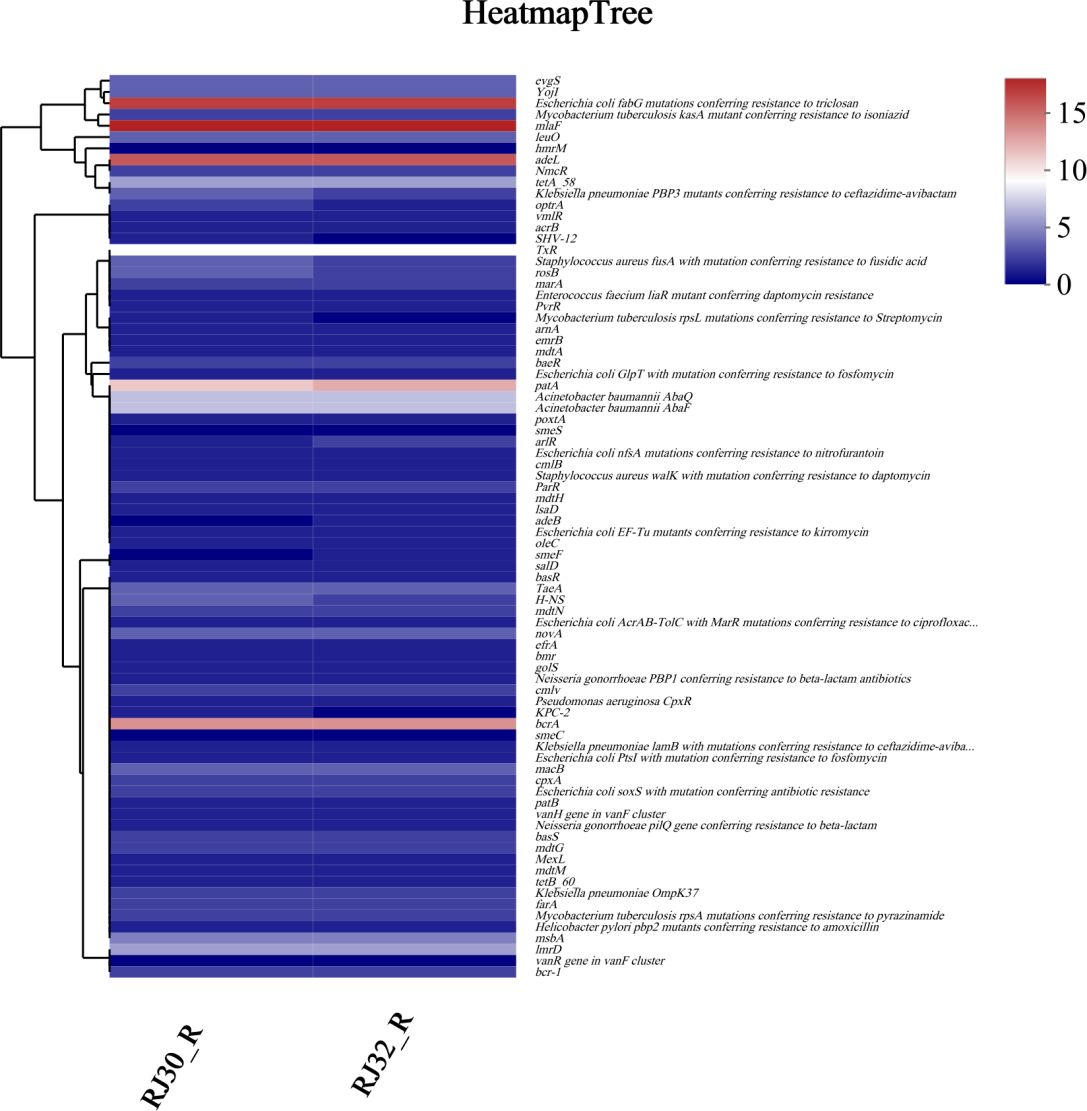
Heatmap of antimicrobial resistance gene clustering. Color blocks represent the relative abundance of resistance genes based on normalized read depths. Red indicates high relative abundance, while blue indicates low relative abundance. The clustering tree is shown on the left side.

Using ResFinder 4.5.0 for resistance gene prediction combined with CARD database alignment for gene characterization, multiple classes of resistance genes were detected in the genomes of both RJ30_R and RJ32_R, primarily involving tetracyclines (*tet* series), sulfonamides (*sul* series), β-lactams (*bla* series), aminoglycoside-modifying enzymes (*aadA*), as well as *ramR* and *acrR*, which are known to regulate efflux pump activity, which are known to confer resistance to their respective antibiotics. Heatmap analysis (Fig. 7) revealed a high relative abundance of these genes, as determined by sequencing coverage and normalized read depths, with significant co-occurrence patterns suggesting a potential linkage between resistance genes. For example, *blaKPC-2* and *bcrA* clustered closely in the same group in the cluster analysis, indicating their frequent co-existence. These results suggest that these resistance genes likely play significant roles in the formation and dissemination of resistance, as evidenced by the observed co-occurrence patterns, which may facilitate the spread of resistance within bacterial populations. However, gene expression levels and functional activities were not validated in this study due to the lack of transcriptomic or functional assays.

### Virulence gene prediction

**Fig 8:**
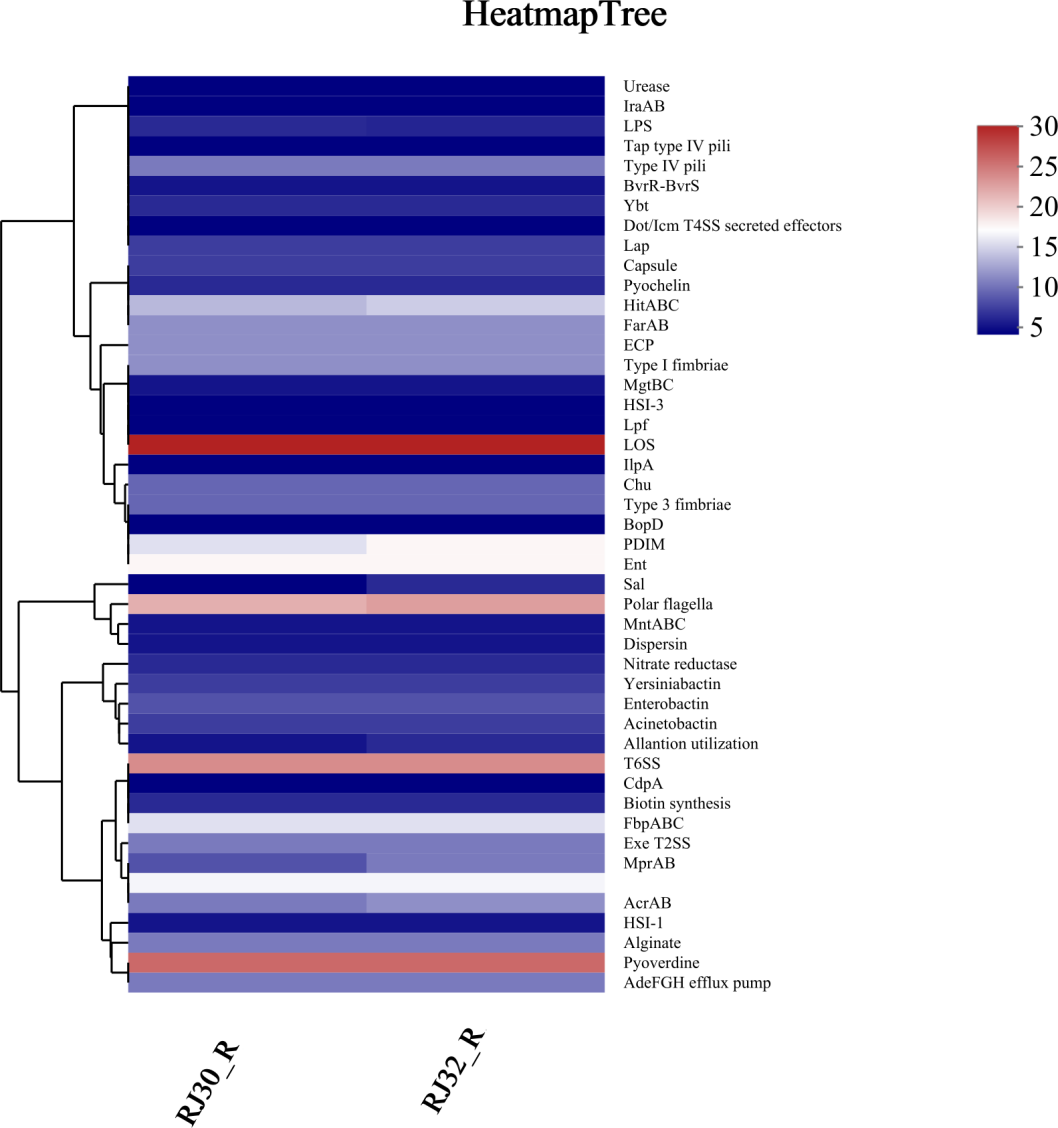
Hierarchical clustering heatmap of predicted virulence genes. Analysis based on VFDB database comparison showing the distribution patterns of virulence factors. Color coding is as in Fig. 7.

Based on VFDB alignment results (Fig. 8), both RJ30_R and RJ32_R were predicted to carry multiple virulence-related genes, which are essential for bacterial pathogenicity and virulence factor identification. Cluster analysis revealed that some virulence factors exhibited co-occurrence patterns with efflux pump genes (such as *AcrAB*) in gene distribution, suggesting a possible genetic linkage between resistance and virulence traits that may facilitate the survival and pathogenicity of these strains. However, classical hypervirulence marker genes (such as *rmpA*, *iucA*, and *iroB*), which are associated with increased pathogenic potential in KP, were not detected in this study, indicating that the actual pathogenic potential of these strains requires further validation through animal models or cell experiments, as these *in vivo* or *in vitro* studies are essential for assessing the virulence and infectivity of the strains under physiological conditions.

### Plasmid typing analysis

**Fig 9:**
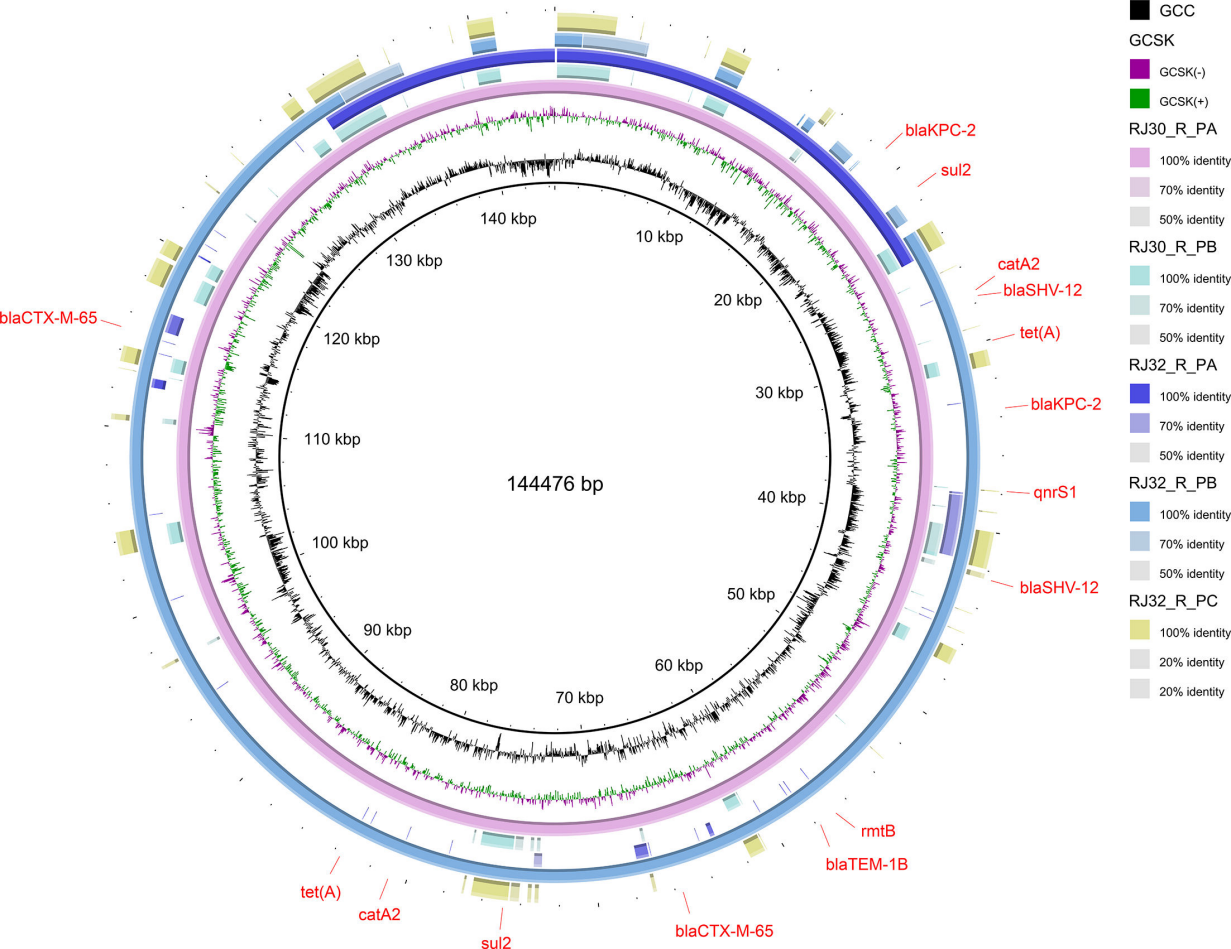
Plasmid circos genome map. This figure shows a circos plot depicting the plasmid profiles of KP strains. The plot visualizes the presence and distribution of key resistance genes, including *blaKPC-2*, *blaSHV-12*, *qnrS1*, *blaTEM-1B*, *blaCTX-M-65*, *rmtB*, *sul2*, *catA2*, and *tet*(A), which are associated with resistance to carbapenems, cephalosporins, fluoroquinolones, aminoglycosides, sulfonamides, and tetracycline. The diagram highlights the gene arrangement on the plasmids, demonstrating the genetic diversity and potential horizontal gene transfer among the strains.

Genome sequencing and sequence alignment results showed that RJ30_R carried two large plasmids, designated PlasmidA and PlasmidB (Fig. 9). Both plasmids exhibited multiple replicon structures, meaning that each plasmid carried more than one replication origin, with PlasmidA (corresponding to contig000020) containing both IncR_1 and IncFII(pHN7A8)_1 replicon types on the same plasmid. This “co-localization” phenomenon, where multiple resistance gene types are found on the same plasmid, is typically considered an important marker of resistance gene integration platforms, which facilitate the co-occurrence and horizontal transfer of resistance genes. Further BLAST alignment showed that PlasmidA had extremely high homology with the reported KP plasmid pKP20194f-p2 (Accession No. NZ_CP054722.1), suggesting a close genetic relationship, while PlasmidB was nearly identical to plasmid pXH1507-3 (Accession No. NZ_CP092796.1), further confirming the presence of common resistance plasmids in these strains. In RJ32_R, three plasmids (PlasmidA, PlasmidB, and PlasmidC) were identified, showing a more diverse plasmid composition. Replicon type analysis revealed that these plasmids contained IncR, IncFII(pHN7A8), repB_KLEB_VIR, and IncHI1B(pNDM-MAR) types, which are commonly associated with MDR gene dissemination across different bacterial strains, contributing to the spread of resistance in clinical settings. The plasmid guanine-cytosine content ranged from 50.09% to 53.86%, consistent with typical *Klebsiella* genus plasmid composition, indicating the normal structural characteristics of these plasmids. Notably, multiple plasmids were enriched in resistance-related coding sequence regions, as categorized by Cluster of Orthologous Groups functional classification, with these regions distributed adjacent to replicon-type genes. These characteristics suggest that these plasmids not only carry multiple resistance genes but also play significant roles in horizontal transmission among clinical strains.

## DISCUSSION

This study confirmed the presence of Tgc-HR phenotypes in clinical CRKP isolates. Through Kirby-Bauer disk diffusion and E-test assays, some isolates exhibited typical scattered colony growth within inhibition zones, which is a characteristic feature of heteroresistance, indicating the presence of resistant subpopulations within an otherwise susceptible bacterial population. When single colonies were selected from within these inhibition zones for secondary susceptibility testing (to confirm the stability and persistence of the heteroresistant phenotype), subclone inhibition zone diameters further decreased, with MIC levels falling within the resistant range and no scattered colonies present within the zones, further supporting the selective amplification of the heteroresistant subpopulation. This phenomenon is consistent with previous reports describing the selective amplification and stable expression of heteroresistant subpopulations under drug selection pressure, where resistant subpopulations are able to proliferate while the susceptible bacteria are inhibited (5, 6, 16). Under conditions without drug pressure, Tgc-HR subclones showed no significant differences in growth curve kinetics (the rate of bacterial growth over time) compared to the parent strains, suggesting that this resistance phenotype may incur relatively low fitness costs. This stability facilitates persistence in clinical and hospital ecological niches, where resistant subpopulations can survive and proliferate, potentially leading to an increased risk of treatment failure, consistent with previous reports of Tgc-HR phenotypes being maintained long-term without selection pressure (5).

Molecular typing results showed that the study strains belonged to ST-11 clones, which are known to be highly prevalent and contribute to the long-term predominance of CRKP in China, consistent with previous epidemiological data (14). The long-term co-evolution between ST-11 and multiple replicon resistance plasmids, where plasmids and bacterial strains evolve together, is believed to promote the widespread dissemination of resistance genes, such as *blaKPC* (17). The detected *IncFII(pHN7A8*) and *IncR* replicon types, along with the high homology between plasmid contigs and reported high-risk plasmids, support their potential role as platforms for the integration and horizontal transfer of multiple resistance genes, as the high homology indicates a shared evolutionary history and potential for gene transfer (18, 19). In this study, we also report 629-locus cgMLST assignments (Kp1/CG11/SL258) to contextualize lineage; however, allelic-distance or single nucleotide polymorphism (SNP)-based phylogenomics for transmission mapping was not performed. Genome annotation results suggested that the strains simultaneously carried multiple classes of resistance genes, including tetracyclines (*tet* series), β-lactams (*bla* series), sulfonamides (*sul* series), and aminoglycoside-modifying enzymes (*aadA*), which collectively contribute to the MDR phenotype and complicate treatment strategies. Among these, plasmid-borne *tet*(A) genes and their variants have been confirmed in previous studies to be closely associated with reduced tigecycline susceptibility, likely due to alterations in the drug’s target binding sites or enhanced efflux mechanisms (13, 14, 20). Since this study did not perform transcriptional level detection or efflux pump functional experiments, we cannot directly confirm whether these genes are highly expressed or active in our isolates, which are crucial steps for determining the direct contribution of these genes to the resistance phenotype. Additionally, mobile RND efflux systems, such as *tmexCD-toprJ*, can confer high-level tigecycline tolerance by actively pumping out the antibiotic, and these systems often spread on plasmids like *IncHI1B*, facilitating horizontal gene transfer and warranting focused monitoring (21, 22).

Chromosomally mediated efflux pump upregulation is also an important mechanism of tigecycline resistance, particularly involving the AcrAB-TolC and OqxAB complexes, which actively pump out the drug and reduce intracellular concentrations, thereby contributing to resistance (23–25). Mutations in regulatory genes, such as *ramR/ramA* and *acrR*, which control the expression of the efflux pumps, can significantly increase pump activity and elevate the MICs, leading to enhanced resistance (23, 24). In our genomic analysis, we identified the presence of *ramR* and *acrR* genes, which are involved in regulating efflux pump activity and are critical to the resistance profiles observed in RJ30_R and RJ32_R. However, given the limited sample size and the lack of transcriptomic analysis and efflux pump inhibition experiments, further studies are needed to validate these mechanisms and gain a deeper understanding of the genetic and functional basis of tigecycline resistance.

Virulence factor analysis predicted that both strains contained multiple virulence determinants (such as toxins, adhesins, and immune evasion factors) and secretion system gene clusters, which are essential for bacterial pathogenicity and host interaction. Co-occurrence patterns were observed alongside *AcrAB* efflux pump genes in the gene distribution, suggesting a potential link between resistance and virulence mechanisms that may enhance bacterial survival and pathogenicity. Although classical hypervirulent KP (hvKP) typically possess marker genes such as *rmpA/rmpA2*, aerobactin, and yersiniabactin, which are associated with enhanced virulence and immune evasion, recent reports of ST-11-KL64 type CR-hvKP suggest that MDR and hypervirulence may co-exist in some lineages, raising concerns about the clinical management of such strains due to their increased pathogenic potential and resistance to multiple antibiotics (17, 26). Therefore, future studies should include screening for typical hvKP markers and incorporate *Galleria mellonella* or mouse models for validation, as these animal models are valuable tools for assessing the virulence and pathogenic potential of bacterial strains in a living host.

This study has several limitations, including a small sample size from a single center, which may not fully represent the national CRKP Tgc-HR epidemiological characteristics, limiting the generalizability of the findings across diverse geographical regions. The results were primarily based on genomic analysis and sequencing data predictions; although multiple resistance genes, virulence factors, and plasmid types were detected, the lack of transcriptomic, quantitative PCR, and efflux pump functional validation prevents the direct determination of actual gene expression levels and functional activities, which are crucial for confirming the functional role of these genes in resistance mechanisms. Plasmid conjugation transfer experiments were not performed, so inferences about resistance and virulence gene transmission risks cannot be conclusively made without further investigation. Future research should expand sample sources, including diverse populations from multiple regions and geographical coverage, incorporating multicenter epidemiological surveys and functional validation to further elucidate the molecular mechanisms and clinical significance of Tgc-HR. Finally, while we now provide cgMLST assignments to contextualize lineage (Kp1/CG11/SL258), we did not perform allelic-distance or SNP-based phylogenomics for transmission mapping. Such analyses will require expanded sampling and a study specifically powered for epidemiological inference.

## Data Availability

The raw whole-genome sequencing data generated in this study have been deposited in the NCBI Sequence Read Archive (SRA) under the BioProject accession numbers PRJNA1327554 (RJ30_R) and PRJNA1327556 (RJ32_R). The corresponding SRA run accession numbers are SRR35934535 and SRR35934402, respectively.
